# My journey into the birth of plant transgenesis and its impact on modern plant biology

**DOI:** 10.1111/pbi.13319

**Published:** 2020-03-18

**Authors:** Luis Herrera‐Estrella

**Affiliations:** ^1^ Laboratorio Nacional de Genómica para la Biodiversidad (Langebio/UGA) Centro de Investigación y de Estudios Avanzados del IPN Irapuato Mexico; ^2^ Institute of Genomics for Crop Abiotic Stress Tolerance (IGCAST) Texas Tech University Lubbock TX USA; ^3^ Department of Plant and Soil Science Texas Tech University Lubbock TX USA

**Keywords:** transgenic plant, gene transfer, gene expression, Ti plasmid, transit peptide, phosphite

## Abstract

I had the fortune to start my scientific carrier during the early stages of the development of plant transformation in one of the leading laboratories in the field. Here, I describe my personal experience in the laboratory of Marc van Montagu and Jeff Schell, and some important contributions that the group made to the development of the technology to produce transgenic plants. I also briefly summarize the impact of this technology on the development of modern plant biology and in plant molecular improvement.

Box 1 1 **Technical Biography**

Luis Rafael Herrera‐Estrella is recognized worldwide for his pioneering work on *Agrobacterium*‐mediated plant genetic transformation and his influential contributions to our understanding of gene expression in crops coping with a continuously changing, often stressful environment. Luis’ contributions have been recognized by numerous awards and distinctions over the years, including the 2002 National Prize from the Government of Mexico, the Trieste Science Prize in 2007, the 2008 Leadership in Science Public Service Award of the American Society of Plant Physiologists and a nomination in 2015 as one of the 100 most influential people in biotechnology by the popular science magazine Scientific American. Luis is an elected member of the Latin American Academy of Sciences, of the Mexican Academy of Sciences, of the Third World Academy of Sciences, of the Academy of Medicine, Engineering and Science of Texas and of the U.S. National Academy of Sciences. He currently is President’s Distinguished Professor of Plant Genomics and Director of the Center for Functional Genomics of Abiotic Stress at Texas Tech University in Lubbock, Texas. For many years, he served as the Director of the National Laboratory of Genomics for Biodiversity at the Center for Research and Advanced Studies of the National Polytechnic Institute in Guanajuato, Mexico, where he now holds the position of Professor Emeritus.

## Introduction

After finishing an M.Sc. degree in genetics and molecular biology at the Center for Research and Advanced Studies (Cinvestav) in Mexico City at the end of 1980, I decided to pursue a scientific career. Under the guidance of a great Mexican microbiologist, Dr. Jose Ruiz‐Herrera, I worked on the influence of light on cell wall biosynthesis in the phototropic response of the fungus *Phycomyces blakesleanus*. However, I was inclined to work on more applied aspects of biology and had chosen the molecular biology of antibiotics biosynthesis as a potential area to pursue my doctoral studies. But I was quickly discouraged by some scientists at Cinvestav telling me that there was no future for such research in Mexico because it was impossible to compete with research groups working at universities or the private industry in developed countries. Many years later, I realized that while it is always hard to compete with groups in developed countries, it is certainly possible to successfully compete in the international arena and to do creative, innovative science in a developing country.

Being discouraged from my first option, I decided that plant biology was a promising field of research since there are in Mexico a large number of native crops that are biologically interesting but of no major importance for American or European seed companies. My knowledge on plants, however, was minimal as I had never taken a course of botany or plant biology; therefore, I had little idea as to what the research trends in plant biology were at that time. While starting to learn about plants, I heard about Dr. Francisco ‘Paco’ Bolivar, one of the postdocs at Herb Boyer’s laboratory, who developed the first plasmid to do recombinant DNA (the old guard will remember the pBR322 plasmid). Dr. Bolivar had just came back to Mexico City to work at the National Autonomous University of Mexico (UNAM). Although I knew little about the topic, I felt that learning more on the recombinant DNA technology would be useful in any area of biology that I would work on later. I asked the general director of Cinvestav to help me contact Paco Bolivar and join his laboratory while I got accepted in a plant biology programme to do my Ph.D. In November 1980, I joined Paco’s laboratory to learn the basics of recombinant DNA technology, including the use of Bal 31, an exonuclease that would become important during my Ph.D. thesis.

At the beginning of 1981, Prof. Marc van Montagu, a leader of the *Agrobacterium* group at University of Ghent, was on his way back to Belgium after a meeting on plasmids in Jamaica. He stopped in Mexico City to give a seminar at UNAM, which I attended without any expectation as I did not know much about *Agrobacterium tumefaciens*. During his talk, I learned about the unique property of this bacterium to transfer plasmid DNA into the chromosomes of plant cells, and how the system was about to initiate a new age of plant genetic engineering. I was truly electrified, indeed, much like when as a four‐year‐old boy I heard for the first time ‘Good Vibrations’ by the Beach Boys or ‘She is a Rainbow’ by The Rolling Stones! (Box 1).

From then I knew that I wanted to work on the genetic modification of plants… But how could an unknown young Mexican student get accepted in the famous laboratory of Marc van Montagu and Jeff Schell in Belgium? I did not know how to approach the problem and was not confident enough to ask Dr. Bolivar to help me contacting them since I had been in his laboratory for just a couple of months. I was also nervous to directly approach Prof. van Montagu as my English was terrible – it still is – and because I did not even know what to say anyway.

At that moment, my nerves had a physiological impact on me and I had to visit the washroom… To my surprise, while entering the washroom, I bumped into Prof. van Montagu who was washing his hands. I do not know how, but suddenly I found the courage and the words to ask him directly: ‘Professor van Montagu, can I join your group as a Ph.D. trainee in Ghent?’ After a few seconds of silence that seemed to me an eternity, he responded: ‘Mmmmm… we normally accept foreigners only at the postdoctoral level, but we never had a Mexican in the group. If you get a letter of recommendation from Prof. Bolivar, I will accept you.’

**Figure 1:**
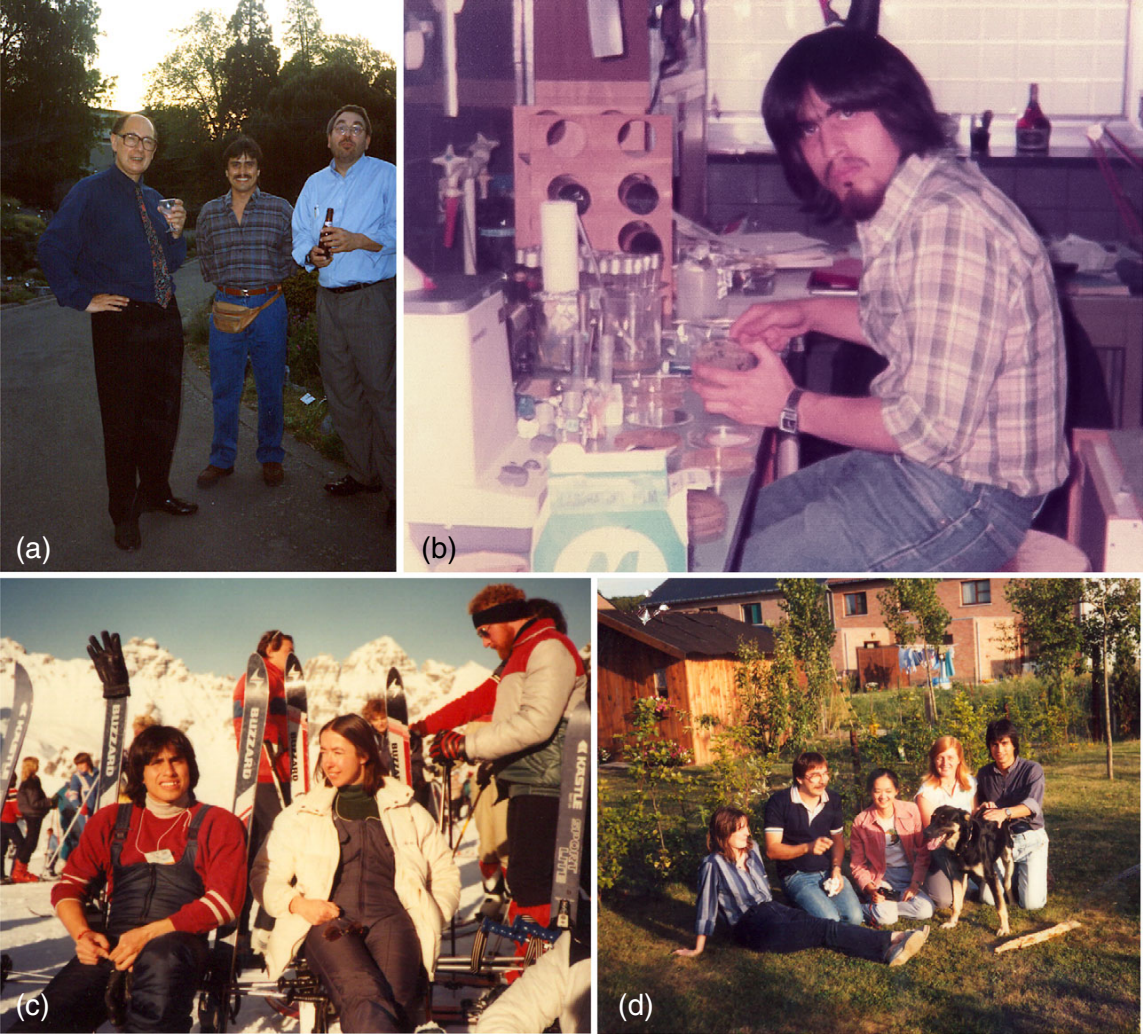
Some pictures of me during my journey in Ghent. (a) With Marc van Montagu and Jan Leemans, drinking beer in the botanical garden of Ghent University. Jan, a former Ph.D. student of Marc, played an important role in the development of insect and herbicide resistance traits at Plant Genetic Systems, a Belgium plant biotech company. (b) Working hard making my deletions of the Nopaline Synthase promoter. (c) Learning to ski with Patricia Zambryski at the Austrian Alps, shortly after we succeed in expressing bacterial genes in tobacco cells. (d) Ready to party! From left to right: June Simpson, Jan Leemans, Kan Wang, Ann Thomas and myself.

I requested the letter, I got it, and in a few weeks, I was accepted, fortuitously and without fully realizing what happened, into one of the best plant/microbiology laboratories in the world! This laboratory was located in a country of which I had little knowledge – although I remembered that our Soccer national team had beated their team 1‐0 in the 1970 World Cup in Mexico – and I had absolutely no clue about Flemish, the Dutch dialect spoken by people in Flanders. Later, I learned that Marc tended to recruit students and collaborators looking more for interesting characters than for their academic talent, which in the end, I think, payed off quite well in my case (Figure 1a).

In June 1981, I left Mexico for my first international trip, a journey to Belgium with, before, a summer stop in London to improve my English. While in London, I spent a fair amount of time reading papers about *Agrobacterium* and T‐DNA transfer. I realized then that I had taken a good decision to do a Ph.D. in Belgium, as this group in Ghent was probably the best in the world on that topic. I landed in Brussels in September 1981 and stayed for the first weekend in the house of a famous Chilean, Nino Villaroel. On Monday, after a busy weekend visiting salsa places, I finally reached the fifth floor of the Ledeganckstraat building of Ghent University, where the Schell/van Montagu laboratory was located. I went to Marc’s office with the intention of expressing my desire to work on developing an expression vector for plant cells, but I was quickly stopped as Marc told me that I had first to learn about *Agrobacterium.*

He assigned me to work with Marcelle Holsters, a smart Belgian scientist there, working on characterizing the border junctions of the DNA fragment transferred from *Agrobacterium* to the chromosomes of plant cells. Marcelle asked me whether I knew how to make Southern blot hybridizations. Although my experience with that increasingly important technique was limited to one Southern blot with *E. coli* DNA, I quickly replied: ‘Yes, of course I do.’ Marcelle asked me to perform a Southern blot analysis of genomic DNA of a tobacco crown gall tumour using as probes Ti plasmid restriction fragments containing the T‐DNA border sequences. Because nomenclature used at the time to name the different regions of the Ti plasmid was based on the size of restriction enzyme fragments, I actually was not clear which exact regions covered the HindIII 23 and EcoR1 15 fragments that I had to use as probes for the Southern blot…

On my first Saturday in Ghent, I did the requested Southern blot hybridization with the expert advice of Nino and left the blot exposing to an X‐ray film for 48 hours. On Sunday morning, I was invited by Marc for a brunch and went with Patricia Zambryski and Allan Caplan, two postdocs in the laboratory, to Marc’s house in Brussels. We had a nice brunch cooked by Marc himself, and I met Nora van Montagu and Lawa, the ocelot they had as pet. Here is the only negative aspect of the brunch: I was offered aged blue cheese at the end of the meal, which for the taste of a ‘gastronomically uneducated’ Mexican used to eat hot Mexican dishes, was the most disgusting food on earth! This brunch could have been irrelevant to my story, but it was actually a significant moment as on the way back to Ghent I could talk with Patricia and Allan of my interest of designing a vector to express foreign gene in plants, and of my wish to find a promoter that would be functional in plant cells. Patricia quickly replied that the nopaline synthase gene of *Agrobacterium*, which upon transfer is expressed in plant cells, had been recently sequenced in the laboratory of Howard Goodman, a collaborator of Marc and Jeff with whom she was working as a postdoc before joining the laboratory in Ghent. Thus, the last piece I needed to develop my expression vector was already available.

Early in the morning on Monday, I developed my autoradiogram to see the results of the crawn gall hybridization. To my surprise, I had generated clear and nice hybridization bands, but that meant little to me. Soon after I developed the film, Allan stopped by and asked me what the hybridization was. He inquired about the probes I had used and immediately concluded that because the probes represented the ends of the DNA fragment transferred from *Agrobacterium* to plant cells and since both of them were hybridizing to the same bands, the T‐DNA fragment was either inserted as tandem copies linked end‐to‐end in the genome of tobacco tumour cells or present in circular form in the cells. About 10 minutes later, Marcelle came into the laboratory and asked me when I was planning to do the Southern blot she had asked for. I replied that it was already done and showed her the autoradiogram. She asked me what my conclusions were, and, like a parrot, I repeated what Allan had just told me a few minutes earlier. I feel she was highly impressed with my answer as a first‐week Ph.D. student working on a new topic!

I kept working with Marcelle for a few weeks, reminding her every day that we should start constructing a vector based on the promoter of the Nopaline Synthase gene to attempt expressing foreign genes in plants… I think she got tired of my insistence and somehow she convinced Marc and Jeff to let me take the design and construction of an expression vector for plant cells as my Ph.D. project. I moved to a new laboratory, on a bench next to Patricia Zambryski, who would then become one of my most important mentors.

It took me about two months to calibrate the amount of material and time of digestion for the Bal31 exonuclease to produce deletion fragments of the *Nos* gene containing only the promoter and the 5’ untranslated regions (Figure 1b). Here, I must remind younger people that at the time, PCR did not exist and DNA synthesis was limited to small size oligonucleotides… We had, then, to produce precise deletions the hard way… After analysing about 230 clones, I finally found a deletion fragment of the right size, which upon sequencing displayed the desired *Nos* promoter sequence. At that stage, I decided to test three coding sequences to determine whether bacterial genes could be expressed in plant cells: the octopine synthase (OCS) coding sequence from *Agrobacterium*, which was shown earlier to be expressed in plant cells; the coding sequence of neomycin phosphotransferase II (NPTII) from the transposon Tn5; and the coding sequence of chloramphenicol acetyl transferase (CAT) from transposon Tn3. NPTII had been recently reported to be an effective selectable marker for animal cells. CAT could be used as a reporter enzyme for which a simple and sensitive enzymatic assay was available.

As a naive Ph.D. student, I was convinced that my project was unique, that no one else in the world was doing something similar and that I would publish my work in *Nature*, just as Paul Berg’s group reporting the use of NPTII as a selectable marker for animal cells in this journal a few months earlier. I worked very hard with June Simpson, a summer student who helped me analyse hundreds of plasmid minipreps to find the clones for expressing NPTII, CAT and OCS in tobacco cells. By the end of 1982, I had results showing the successful expression of the CAT and OCS coding sequences under the control of the NOS promoter in tobacco cells, and preliminary results in collaboration with Marc De Block showing that NPTII could be used along with kanamycin as a selectable marker system for plant cells. I remember quite clearly, in December 1982, Jeff Schell coming to the laboratory and asking me: ‘How is your project progressing?’ Very proudly, I told him that we had very convincing results in hand showing the expression of bacterial genes in plant cells and suggesting the potential of NPTII as a selectable marker for plant cells. I almost fainted when he told me: ‘Good, very good, because the guys at Monsanto will present their results on the use of the NOS promoter and NPTII as a selectable marker for plant cells next month at the Miami Winter Symposium and I will present your results.’

For the first time, I was hearing about ‘Darth Vader’ Monsanto! I was not the only person working on a project to express foreign genes in plant cells… Many other laboratories had similar projects ongoing, including that team of 10 people at Monsanto! To recover from the shock, I went to Switzerland to spend Christmas with Mexican friends and learn to ski with Patricia (Figure 1c). Coming back to Ghent, I worked day and night to submit my first publication on the expression of bacterial genes CAT and OCS in plant cells. My dream of publishing in *Nature* became reality in the 19 May 1983 issue of the journal (Herrera‐Estrella *et al.*, 1983a). In June of the same year, we published a second paper, in EMBO Journal, about our work on NPTII as a selectable marker for plant cells (Herrera‐Estrella *et al.*, 1983b), a couple of months before the Monsanto team also publish their work on NPTII, in the August 1983 issue of PNAS (Fraley *et al.*, 1983).

We published again in *EMBO Journal* in 2004 to confirm the expression of foreign genes in regenerated plants and their progeny (de Block *et al.*, 1984), the same year as Monsanto presented similar conclusions in a *Science* paper (Horsch *et al.*, 1984). I was not aware at that time of the implication of keeping dated laboratory notebooks for intellectual property rights on discoveries of eventual practical relevance. I did not realize that years later such notebooks would become so important in the legal battle for the intellectual property rights on *Agrobacterium*‐mediated transformation between Monsanto and the laboratories in Ghent, represented by the Max Planck Institute in Cologne where Jeff Schell was then director. The reasons why we lost the rights on these *Agrobacterium* patents after a long and expensive legal battle that lasted over 20 years are still a mystery to me.

The availability of an efficient system for gene transfer to plant cells opened the possibility of studying a wide range of gene regulation and cell biology processes in plant’s but the cloning of plant genes in the mid‐1980s was just emerging. Soon after we publish our work on the expression of foreign genes in plant cells, Anthony ‘Tony’ Cashmore came to Ghent to present a seminar about the cloning and characterization of genes encoding the small subunit of ribulose bisphosphate carboxylase (RBCs) and chlorophyll A/B binding proteins (CAB). These proteins that play important roles in light harvesting and CO_2_ fixation during the photosynthetic process were known to show tissue‐specific and light‐regulated expression patterns. Immediately, we started a collaboration with Tony, then a professor at the Rockefeller University in New York City, to determine whether the sequences mediating tissue‐specific and light‐inducible expression of the RBCs gene resided in its 5’ flanking sequence. In 1984, we published this work in *Nature*, reporting that the RBCs promoter was capable of directing the expression of the CAT coding sequence in a light‐dependent and tissue‐specific manner (Herrera‐Estrella *et al.*, 1984). This was the first report of a functional plant promoter successfully expressed in transgenic plant cells.

Later, we worked with Mike Timko, then a postdoctoral fellow at the Rockefeller University, to uncover a first transcriptional enhancer in plants, also part of the RBC gene 5’ flanking sequence (Timpko at al., 1985). During the same period, June Simpson described a transcriptional enhancer/silencer in one of the CAB gene promoters and showed all signals, chloroplast retrograde, phytochrome‐dependent and tissue‐specific, were converging to target the promoter of RBC and CAB genes and regulate their expression (Simpson *et al.*, 1985; Simpson *et al.*, 1986). Years later, two of my students in Mexico, Gerardo Argüello and Aida Martínez, showed that a minimal element composed of an I‐box and a G‐Box was sufficient to control light‐inducible, tissue‐specific and phytochrome‐dependent gene expression (Martínez‐Hernández at al., 2002). Still as part of the group in Ghent, together with Guido van den Broeck and in collaboration with Tony Cashmore, Mike Timko and Albert Kausch from Rockefeller, we did elegant work to demonstrate that the transit peptide of nuclear‐encoded proteins was sufficient to target heterologous proteins into the chloroplast (van den Broek, *et al.*, 1985). The use of transit peptides for the import of foreign proteins into chloroplasts was patented would become an essential tool for the development of the glyphosate‐resistance technology commercialized by Monsanto.

I also had the good fortune, during this period, to work with Teemu Teeri to develop the first promoter trap system for plant cells (Teeri *et al.*, 1986) and with Kan Wang and Patricia Zambryski to determine that T‐DNA borders had vectorial activity in transferring DNA into plant cells (Wang *et al.*, 1984). We were working very hard, but we were also enjoying life, often going with June, Teemu, Kan and Guido for a ‘terminal beer’ at one of those several nice cafes we could find in Ghent (Figure 1d). Everything happened very fast, and we were not truly realizing the importance of our work for the future of plant biology. By the end of 1986, we had published ten papers in major journals that represented, after all, only a fraction of the papers published by the Schell/van Montagu group during these years.

After a fantastic time in Ghent, and in spite of receiving job offers from a couple of companies and prestigious universities in Europe and the Unites States, I decided returning to Mexico. Upon my arrival, I was asked to organize a new department at the Center for Research and Advanced Studies (Cinvestav) dedicated to plant genetic engineering. During my first five years in Mexico, I focused on the development of transformation systems for crops important to Mexico, but with mixed success. Transforming amaranth, tomatillo (*Physallis ixocarpa*) or papaya was easy, but transforming common bean and chilli pepper proved to be quite difficult and only led to sporadic results that we never published. My group then moved on studying different types of abiotic stresses, including drought and nutritional stress. We made a number of interesting contributions to the field, of which I will here mention only a couple due to space limitation.

One important, but controversial, paper has been the one in which we reported that expressing a bacterial citrate synthase could enhance aluminium (Al) tolerance in tobacco and papaya (De la Fuente *et al.*, 1997). Al is an abundant but inert metal in most soils, which in acidic soils becomes soluble and highly toxic to most plant species. A natural mechanism of tolerance to Al in acidic soils is the release of organic acids that chelate and prevent the entry of Al into the roots. In our hands, the expression of a citrate synthase from *Pseudomonas aeruginosa* increased tolerance to Al *in planta*, but later a second group working on the *E. coli* citrate synthase found no such tolerance. Fortunately, several publications soon after showed that the expression of bacterial citrate synthases or the overexpression of plant citrate synthases could indeed confer increase Al tolerance in crops. We also showed that phosphate (Pi), an essential nutrient for plants, could act as a signal to trigger plant responses to low Pi availability. Before our publication on *Arabidopsis* mutants insensitive in the root architecture response, whether plant responses to Pi deficiency were only due to metabolic stress or to a specific role for Pi as a signalling molecule was still a controversial question.

Perhaps the most ingenious piece of research work to come out of my laboratory in Mexico was the development by Damar López‐Arredondo of a system of crop selective nutrition to reduce the use of phosphorus (P) fertilizers and herbicides in agricultural fields (López‐Arredondo and Herrera‐Estrella, 2012). Most organisms can only use Pi as a nutritional source of P and are therefore unable to metabolize other chemical forms of this essential element. Phosphite (Phi), a reduced form of P, has been used in agriculture for several decades as a fungicide because it inhibits fungal growth and enhances the natural defences of plants. As plants are unable to metabolize Phi, farmers need being careful not to add excessive amounts of Phi as it can inhibit plant growth by competing with Pi for entry in root tissue and interfering with several biochemical reactions. Our idea was simple: we knew that some bacteria could metabolize Phi using an oxidoreductase to convert it into Pi to support metabolism. We thought that if we could engineer plants to use Phi as a P source, we could selectively fertilize our favourite crop with Phi while at the same time inhibit the growth of weeds unable to metabolize this compound in low‐Pi soils. This system proved to be quite robust and to work well both as a selectable marker for plant transformation and as an alternative P nutrient source for the selective fertilization of tobacco, cotton, rice, maize and soybean. The technology has the potential to reduce the amount of P, a non‐renewable resource, for crop nutrition in agriculture. It also has the potential of reducing the use of herbicides, which consumer increasingly dislike. Implementing it in agricultural fields, however, still remains challenging given current and growing public concerns about the use of genetically modifies crops, even though the engineered plants have the potential of reducing the environmental footprint of agriculture.

Last but not least, I would like coming back to those comments of fellow Mexican colleagues about the impossibility, in less developed countries, of doing research of sufficient quality to publish in high‐impact journals. Work done by my research group in Irapuato, a small town in the Guanajuato State, has generated over the years two publications in *Science*, one in *Nature*, two in *Nature Biotechnology*, three in *The Plant Cell* and several in *PNAS*. It is possible, indeed, to publish in high‐impact journals while working in countries with limited support for research and few colleagues with whom to collaborate. For this, we need to read more, to remain creative and to always make the best use of the limited resources we have access to.
